# Italian cross-cultural adaptation of the EveryONE Social Needs Screening Tool of social determinants of health in primary care

**DOI:** 10.1017/S1463423625100418

**Published:** 2025-09-04

**Authors:** Lorenzo Campedelli, Lucia Palandri, Viviana Forte, Vanessa Eugenia Privitera, Peter Konstantin Kurotschka, Giulia Ugolini, Silvia Riccomi, Francesca Rossi, Silvia Keeling, Cinzia Scauri, Elena Righi, Alice Serafini

**Affiliations:** 1 Department of Primary Care, Local Health Authority of Bologna, Bologna, Italy; 2 Section of Public Health, Department of Biomedical, Metabolic and Neural Sciences, University of Modena and Reggio Emilia, Modena, Italy; 3 Department of Medical Sciences and Public Health, University of Cagliari, Cagliari, Italy; 4 Department of General Practice, University Hospital Wuerzburg, Wuerzburg, Germany; 5 Department of Primary Care, Local Health Authority of Modena, Modena, Italy; 6 Department of Biomedical, Metabolic and Neural Sciences, University of Modena and Reggio Emilia, Modena, Italy; 7 Laboratorio EduCare, University of Modena and Reggio Emilia, Modena, Italy; 8 Scuola Normale Superiore, Firenze, Italy; 9 Social Service, Minor Protection Area of the Municipality of Bologna, Bologna, Italy

**Keywords:** cross-cultural adaptation, Italy, primary care, screening, social determinants of health

## Abstract

Social disadvantage can result in healthcare gaps and primary care may be a suitable healthcare context to identify unmet social needs. A variety of screening tools exists but none of them is consolidated in clinical practice. After reviewing the available instruments, we conducted a rigorous translation and trans-cultural adaptation into Italian language of the EveryONE social need screening tool questionnaire of the American Academy of Family Physicians. The translated questionnaire was piloted among 45 patients consecutively recruited in two general practices in the northern Italian city of Modena in 2023 and obtained excellent scores in comprehension and acceptability. The cross-cultural adaptation presented in this study is a first step towards a complete validation. A full validation study is needed to safely adopt EveryONE in routine general practice and to evaluate its effects on health provision.

## Introduction

Social determinants of health (SDoH) generally refer to ‘any nonmedical factors influencing health, including health-related knowledge, attitudes, beliefs, or behaviours’ (Braveman *et al.*, 2011) and include the environments where people are born, live, work, play, worship (WHO, 2008).

Social disadvantage and inequality can affect morbidity and mortality. Therefore, health institutions are becoming increasingly involved in SDoH research. The WHO Commission on determinants of health described how to tackle health inequity through action on SDoH (WHO, 2008). One approach is to integrate them into clinical workflows and decision-making processes so computer models have been built to pursue the goal of collecting and documenting individual-specific social risks and help organizations fill technical, operational, and policy gaps (Espinoza *et al.*, 2023). There is, however, a clear need to customize SDoH screening tools to local needs (LaForge *et al.*, 2018).

Primary Care (PC) is a relevant healthcare context to identify unmet social needs and to connect patients with the community resources to overcome them (Marmot and Bell, 2012). Equity of care, person-centred care and community-oriented care are core values of every general practitioner (GP) (Forte *et al.*, 2023; Wonca Europe, 2023), as GP practices represent ideal settings for implementing and supporting screening programmes of SDoH, due to their regularity of contact and better continuity of care with patients compared to other specialist or emergency care settings (van Doorslaer *et al.*, 2006; Boch *et al.*, 2020). Despite this, a recent systematic review shows how most of the needs detected were unknown to physicians (Page-Reeves *et al.*, 2016; Wilhite *et al.*, 2020; Novilla *et al.*, 2023) and despite SDoH screening is perceived as an important issue, adopting it into the clinical workflow can be difficult (Gruss *et al.*, 2021).

In addition, considering the Italian context, validated screening instruments in Italian to detect social needs in healthcare settings are missing. To fill this gap, we reviewed existing screening tools, possibly designed for the PC context, to perform a cross-cultural adaptation into Italian language

## Aims

To identify a suitable Social Needs Screening Tool for PC setting and to translate and interculturally adapt it to the Italian context; to develop a reliable, comprehensible and acceptable questionnaire for patients.

## Methods

### Study setting

The pilot test was conducted from 22 May to 18 June 2023, in two associated Family Medicine practices in which worked a total of 9 GPs, already involved in research in PC and deprived populations (Di Biagio *et al.*, 2019; Serafini *et al.*, 2023; Ugolini et al., 2023; Veronesi *et al.*, 2019). The two practices were comparable in terms of size of assisted population (nearly 5000 patients) and located in two similar neighbourhoods of Modena (Emilia-Romagna, Northern Italy), a city with a low level of social deprivation. The tool was entirely self-administered and offered in the waiting room to all patients presenting to the practices from a GP trainee who briefly explained its purpose and asked patients if they were interested in participating, clarifying that they were not required to answer the questions, but only to evaluate their comprehensibility and acceptability. The inclusion criteria for the convenience sample were: age over 14 years, willingness to participate. Exclusion criteria were: severe cognitive impairment and severe language barrier. Participants rated the clarity of the items using a five-point Likert scale (from 0 = ‘Not at all clear’ to 5 = ‘Perfectly clear’), and their acceptability by answering to a dichotomous question (yes/no) for each item. Those who rated an item as unclear were asked to provide suggestions on how to rewrite it to make the language clearer. As per validation guidelines, changes in items would be implemented if at least 20% of the sample considered the item unclear (Topf, 1986; Sousa and Rojjanasrirat, 2011)

### Ethics aspects

This study was conducted in accordance with the Declaration of Helsinki. This study did not require the approval of an ethical committee because the questionnaire data were anonymous, making it impossible to identify and harm any respondent. Moreover, neither drugs nor medical devices were prescribed/administered. As a result, the responses were collectively examined while taking into account Italian and European regulations governing the management of personal data.

### Questionnaire integration

As an adaptation to the Italian context, the multidisciplinary expert committee, based on the direct experience with patient needs not covered by the original tool, decided to investigate four more domains, adding five items to the original instrument. These additions were informed by prior validated instruments, professional experience, and input from a patient representative and social workers. The goal was to enhance contextual relevance for the Italian setting, particularly regarding health system structure and prevalent social issues. The following domains have been investigated with additional items:

a) Responsibility as caregiver: an item was adapted from the BMC-THRIVE questionnaire (Buitron de la Vega *et al.*, 2019); b) Social support: two items were added: one was adapted from the Medicare Total Health Assessment Questionnaire (Kaiser, 2012), another from the report by Thayer (Thayer and Anderson, 2018); c) Accessibility of health care: a specific item was developed for the Italian context where insurances have a limited role; d) Immigration status: an item was formulated with the support of the social worker and the patient’s trainer.

### Description of the instrument

To identify a SDoH screening tool suitable for PC settings, we established a multidisciplinary committee of experts composed of two general practitioners, a public health specialist and epidemiologist, a sociologist, a patient representative and patient partner, and a social worker. The group conducted an analysis of various available tools, mainly those selected by two recent reviews (De Marchis *et al.,* 2022; Moen *et al.,* 2020) that performed a review of available SDoH screening tools (Sherin *et al.*, 1998; Basile *et al.*, 2007; Garg *et al.*, 2007; Hager *et al.*, 2010; Brcic *et al.*, 2015; Page-Reeves *et al.*, 2016; American Academy of Family Physicians, 2018; Mahalingam *et al.*, 2020). After reviewing the available literature we selected the American Academy of Family Physicians (AAFP)’s Social Needs Screening Tool from the EveryONE Project. We chose it for the following reasons: a) the existence of two versions, a long and short one, with the latter resulting useful in time-limited situation such as GP practice b) the health-related social needs explored by the short version: *housing, food, transport, public services and personal safety;* c) the way of administration: it can be self-reported; d) the presence, alongside the SDoH screening, of a services’ map (Neighbourhood navigator) to connect the social needs of patients with the appropriate services, as it is not considered ethical to screen needs without appropriate referral (Kanatli and Yalcin, 2021); e) the suitability for people with medium-low level of education.

#### Translation and cross-cultural adaptation

**Figure 1.**
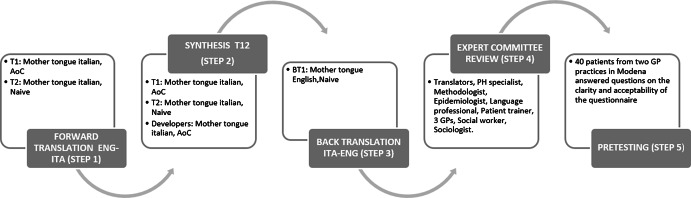
Cross-cultural adaptation process in steps, adapted for Beaton *et al*. Legend: T: translator; BT: back-translator; AoC: aware of the concept; PH: Public Health; GP: general practitioner.

The term ‘Intercultural adaptation’ refers to a process that examines both linguistic (translation) and cultural issues (Beaton *et al.*, 2000). We performed a cross-cultural adaptation process following Beaton’s guidelines (Figure 1). Two independent translators, native Italian speakers, bilingual and bicultural – one (T1) aware of the concepts (AoC) covered with clinical experience and the other (T2) not AoC- translated the original language instrument into the target language (TL, Italian). They produced two independent translations. The following steps consisted of a meeting between developers (native Italian speakers fluent in English, one with experience in cross-cultural translation-adaptation) and translators to analyse the versions, resolve any discrepancy and generate a synthesized version (T12). An additional translator (BT1, native English speaker resident in Italy), blinded to the first version and naive to the concepts, retranslated the questionnaire T12 into the original language (back translation). The same multidisciplinary committee of experts reviewed all previous versions, comparing the original version with the proposed translations according to four areas of equivalence: semantic, idiomatic, conceptual and experiential (Beaton *et al.*, 2000). The multidisciplinary expert committee, after three rounds of online meetings, agreed on the final translation.

#### Statistical analysis

We summarized findings using descriptive statistics: absolute and relative frequency for categorical variables and mean and standard deviation for the continuous variables.

## Result

### The EveryONE social needs screening tool – Italian version

The final version of the screening instrument contains 20 items addressing commonly studied SDoH domains and an open question for possible additional information. The final version is available as a Supplementary file.

### Pilot testing results

45 consecutively recruited patients (from 25/05/2023 to 01/06/2023) attending two different practices in Modena were eligible and only five of them refused to participate. Within the sample, 58% were women, 83% were Italian native speakers and the main age groups (15–35, 36–55, 56–74 years) were quite equally represented excluding the over 75s accounting for only 10%.

#### Comprehensibility

Most participants found the questionnaire clear and comprehensible (Table 1). Most items (18 over 20) received a rate ≥4 (‘Clear’ and ‘Perfectly clear’) on the 0–5 Likert scale by at least 95% of respondents. Two questions (items 12 and 13) resulted slightly more difficult to understand, however, they received a rate ≥4 by 92% and 90% of respondents. Few differences in rating can be observed according to age, gender or mother tongue subgroups, nevertheless they did not result statistically significant (See Table 1). Given that the questionnaire was deemed clear by at least 80% of the samples, no changes were made to the translated manuscript.

**Table 1.** Overall comprehensibility and acceptability and subgroup analysis of The EveryONE Social Needs Screening Tool – Italian version

Understandability (≥ 4 on a 0–5 Likert scale)										*Acceptability (yes)*								
	*Overall*	*Age* *15–35*	*Age* *36–55*	*Age* *56–74*	*Age* *>75*	*Male*	*Female*	Mother Tongue (italian)	Mother Tongue (non italian)	*Overall*	*Age* *15–35*	*Age* *36–55*	*Age* *56–74*	*Age* *>75*	*Male*	*Female*	Mother Tongue (italian)	Mother Tongue (non italian)
	(n:40)	(n:11)	(n:10)	(n:12)	(n:4)	(n:14)	(n:23)	(n:33)	(n:4)	(n:40)	(n:11)	(n:10)	(n:12)	(n:4)	(n:14)	(n:23)	(n:33)	(n:4)
Item																		
1. EDUCATION	40 (100)	11 (100)	10 (100)	12 (100)	4 (100)	14 (100)	23 (100)	33 (100)	4 (100)	40 (100)	11 (100)	10 (100)	12 (100)	4 (100)	14 (100)	23 (100)	33 (100)	4 (100)
2. EMPLOYMENT	37 (92)	11 (100)	10 (100)	9 (75)	4 (100)	13 (93)	21 (91)	30 (91)	4 (100)	39 (97)	11 (100)	10 (100)	11 (92)	4 (100)	14 (100)	22 (96)	32 (97)	4 (100)
3. FOOD	39 (97)	11 (100)	9 (90)	12 (100)	4 (100)	13 (93)	23 (100)	33 (100)	3 (75)	37 (92)	10 (90.9)	8 (80)	12 (100)	4 (100)	12 (86)	22 (96)	30 (91)	4 (100)
4. FOOD	39 (97)	11 (100)	9 (90)	12 (100)	4 (100)	13 (93)	23 (100)	33 (100)	3 (75)	38 (95)	11 (100)	8 (80)	12 (100)	4 (100)	13 (93)	22 (96)	32 (97)	3 (75)
5. HOUSING	37 (92)	11 (100)	8 (80)	11 (92)	4 (100)	13 (93)	21 (91)	32 (97)	2 (50)	39 (97)	11 (100)	9 (90)	12 (100)	4 (100)	14 (100)	22 (96)	32 (97)	4 (100)
6. HOUSING	39 (97)	11 (100)	10 (100)	11 (92)	4 (100)	14 (100)	22 (96)	32 (97)	4 (100)	39 (97)	11 (100)	9 (90)	12 (100)	4 (100)	14 (100)	22 (96)	32 (97)	4 (100)
7. UTILITIES	40 (100)	11 (100)	10 (100)	12 (100)	4 (100)	14 (100)	23 (100)	33 (100)	4 (100)	39 (97)	11 (100)	9 (90)	12 (100)	4 (100)	14 (100)	22 (96)	32 (97)	4 (100)
8. FINANCES	40 (100)	11 (100)	10 (100)	12 (100)	4 (100)	14 (100)	23 (100)	33 (100)	4 (100)	37 (92)	11 (100)	8 (80)	11 (92)	4 (100)	12 (86)	22 (96)	30 (91)	4 (100)
9. TRANSPORTATION	39 (97)	11 (100)	10 (100)	11 (92)	4 (100)	14 (100)	22 (96)	32 (97)	4 (100)	40 (100)	11 (100)	10 (100)	12 (100)	4 (100)	14 (100)	23 (100)	33 (100)	4 (100)
10. ^*^ACCESSIBILITY OF HEALTH CARE	38 (95)	11 (100)	8 (80)	12 (100)	4 (100)	13 (93)	22 (96)	33 (100)	2 (50)	38 (95)	10 (90.9)	9 (90)	12 (100)	4 (100)	13 (93)	22 (96)	31 (94)	4 (100)
11. CHILDCARE	40 (100)	11 (100)	10 (100)	12 (100)	4 (100)	14 (100)	23 (100)	33 (100)	4 (100)	40 (100)	11 (100)	10 (100)	12 (100)	4 (100)	14 (100)	23 (100)	33 (100)	4 (100)
12. ^*^CAREGIVING	37 (92)	11 (100)	8 (80)	11 (92)	4 (100)	12 (86)	20 (87)	30 (91)	4 (100)	39 (97)	11 (100)	10 (100)	11 (92)	4 (100)	14 (100)	22 (96)	32 (97)	4 (100)
13. ^*^SOCIAL SUPPORT	36 (90)	9 (82)	9 (90)	11 (92)	4 (100)	13 (93)	20 (87)	30 (91)	3 (75)	39 (97)	11 (100)	9 (90)	12 (100)	4 (100)	13 (93)	23 (100)	32 (97)	4 (100)
14. ^*^SOCIAL SUPPORT	38 (95)	10 (91)	10 (100)	11 (92)	4 (100)	13 (93)	22 (96)	31 (94)	4 (100)	39 (97)	11 (100)	10 (100)	12 (100)	3 (75)	14 (100)	22 (96)	32 (97)	4 (100)
15. ^*^IMMIGRATION	39 (100)^a^	11 (100)	10 (100)	12 (100)	4 (100)	14 (100)	23 (100)	33 (100)	4 (100)	32 (91)^b^	8 (72.7)	8 (80)	10 (83)	4 (100)	13 (93)	17 (89)	27 (93)	3 (75)
16a. PERSONAL.SAFETY	40 (100)	11 (100)	10 (100)	11 (92)	4 (100)	14 (100)	22 (96)	32 (97)	4 (100)	34 (85)	9 (81.8)	8 (80)	11 (92)	4 (100)	12 (86)	20 (87)	28 (85)	4 (100)
16b. PERSONAL.SAFETY	39 (97)	11 (100)	10 (100)	11 (92)	4 (100)	14 (100)	22 (96)	32 (97)	4 (100)	37 (92)	11 (100)	8 (80)	11 (92)	4 (100)	13 (93)	21 (91)	30 (91)	4 (100)
16c. PERSONAL.SAFETY	39 (100)^c^	11 (100)	10 (100)	12 (100)	4 (100)	14 (100)	22 (96)	32 (97)	4 (100)	38 (95)	11 (100)	8 (80)	12 (100)	4 (100)	13 (93)	22 (96)	31 (94)	4 (100)
16d. PERSONAL.SAFETY	38 (97)^d^	11 (100)	10 (100)	11 (92)	4 (100)	14 (100)	22 (96)	32 (97)	4 (100)	36 (90)	11 (100)	8 (80)	10 (83)	4 (100)	12 (86)	21 (91)	29 (88)	4 (100)
17. ASSISTANCE	38 (95)	11 (100)	10 (100)	11 (92)	4 (100)	14 (100)	22 (96)	32 (97)	4 (100)	38 (95)	11 (100)	10 (100)	11 (92)	4 (100)	14 (100)	21 (91)	31 (94)	4 (100)

* Items added from the original version. Data are presented as *n* (%). ^a,c,d^missing data for one participant, ^b^missing data for 5 participants.

#### Acceptability

The results on acceptability were also positive (Table 1): three questions appeared acceptable by all respondents and 11 by over 95%. Items 15, 16a, and 16d resulted slightly less acceptable, however, they received a positive evaluation by 80% of participants. Further, items regarding intimate-familiar spheres, particularly item 16a and 16d – addressing intimate partner violence (IPV) – were commented with negative feeling (‘*fear’*, ‘*shame’*, ‘*too closed question’)*. Only small and not statistically significant differences were observed across age, gender and native language subgroups.

## Discussion

In the present study we carried out a translation and cross-cultural adaptation to Italian of the original version of the AAFP Social Needs Screening Tool of the EveryONE Project (American Academy of Family Physicians, 2018). We subsequently administered the tool to a sample of 40 patients from two GP practices in Modena to assess its clarity and acceptability. Overall, patients judged the tool to be comprehensible and acceptable. Accordingly, previous research evaluating SDoH screening showed how healthcare professionals felt comfortable with the inclusion of SDoH data in their electronic medical records (EMR) (De Marchis *et al.*, 2019; Mullen *et al.*, 2023). Previous evidence also showed how items related to IPV causes discomfort to a small percentage of the respondents only, while most feel comfortable with these questions (Brown *et al.*, 2000). In the same study, at least 91% of the women reported feeling comfortable or very comfortable when the GP asked questions about alleged violence, however, battered women felt significantly less comfortable than non-maltreated women with questions about physical and sexual abuse. These findings should be considered in the planning of a future proper validation study, in order to build a safe environment to administer the screening tool; in addition, the involvement of an IPV professional should be necessary. Other items (i.e. 2, 11 and 13 – See Table 1), were considered ambiguous by a few responders; however, guidelines stated that changes to the instructions, response format and items of the instrument should be made if at least 20% of the sample members consider it unclear and in our case this percentage was not reached (Topf, 1986; Sousa and Rojjanasrirat, 2011), so we did not change the items. Analysing the answers of the foreign language subgroup, the unclear items were probably related to the language barrier, this could be overcome by instructing all patients not to answer the unclear questions and to wait to clarify them with the doctor during the interview.

### Limitations of the study

First of all, this is a preliminary validation study and a full validation is needed to adopt the EveryONE screening tool in clinical practice, in particular regarding the items that the research team chose to add that were not present in the original version. Another limitation is the potential for selection bias, as the pilot testing was conducted in a single, medium-sized, high-income urban setting. Therefore, the generalizability of the results is limited. Future validation efforts should include a broader range of settings, particularly with greater socio-economic diversity. In addition, the limited number of foreign language-speaking participants can represent a further limit of study.

### Implications for policy and research

To proceed with a full validation of the instrument, several preparatory steps are required. First, ethical approval must be obtained to allow access to patients’ personal data and GP’s medical records. Additionally, the study sample will have to include GPs operating in a wider range of Italian settings, ensuring variability in geographic location and socio-economic context. Finally, prior to implementation, participating GPs should undergo specific training to ensure they are adequately prepared to manage the social needs identified, particularly in cases involving vulnerability or risk, such as domestic violence or severe deprivation. Without such training, the collection of sensitive information could raise ethical concerns, as unmet requests for help may exacerbate patient distress. After a proper validation, researchers could evaluate how to integrate it into GPs’ management software and to understand and measure how this new data flow will influence doctors’ approach. According to literature (WHO, 2008; Marmot and Bell, 2012) this tool could help to activate/coordinate services to tackle social needs, to empower local resources and to strengthen equity challenges. This tool, integrated into the EMR, could produce further insights into correlations between SDoH and healthcare outcomes, problems and behaviours in the specific context of Italian PC services.

## Conclusions

We translated and performed a trans-cultural adaptation into Italian language of the EveryONE Social Need Screening Tool to address social determinants of health in PC. The original questionnaire was integrated with new domains adapted to the Italian context. The tool obtained excellent scores in comprehensibility and acceptability. Health equity is a marker of societal progress and although PC has as its fundamental principle the orientation towards social problems and the holistic approach, sometimes in the routine it can be difficult to monitor how effectively these values are applied. This screening tool could therefore allow GPs to systematically identify social risk factors and therefore orient appropriate care processes.

## Supporting information

Campedelli et al. supplementary material Campedelli et al. supplementary material

## Supplementary material

The supplementary material for this article can be found at https://doi.org/10.1017/S1463423625100418
