# Supplementary material for: Italian cross-cultural adaptation of the EveryONE Social Needs Screening Tool of social determinants of health in primary care
Source: Prim Health Care Res Dev. 2025 Sep 4;26:e76. doi: 10.1017/S1463423625100418 (PMC12455350; doi:10.1017/S1463423625100418)
Supplement: Campedelli et al. supplementary material [file S1463423625100418sup001.pdf]

UNIMORE
UNIVERSITÀ DEGLI STUDI DI MODENA E REGGIO EMILIA

SERVIZIO SANITARIO REGIONALE
EMILIA-ROMAGNA
Azienda Unità Sanitaria Locale di Modena

Questionario per lo Screening dei Determinanti Sociali di Salute (DSS) in Cure Primarie

	↓ **La domanda è chiara?**	↓ **La domanda ti crea disagio?**
	↓ **0 = Per niente chiara**	↓
	↓ **5 = Chiarissima**	↓
1. Hai un diplomaa di scuola superiore?<br>☐ Sì ☐ No	**0** 1 2 3 4 **5**	☐ **No**<br>☐ **Si, perché**<br>....................................
2. Hai un lavoro?<br>☐ Sì ☐ No	**0** 1 2 3 4 **5**	☐ **No**<br>☐ **Si, perché**<br>....................................
3. Negli ultimi 12 mesi, è successo che il cibo a casa tua finisse e non hai avuto soldi per comprarne dell'altro?<br>☐ Si, sempre ☐ No, mai<br>☐ Si, spesso<br>☐ Si, a volte	**0** 1 2 3 4 **5**	☐ **No**<br>☐ **Si, perché**<br>....................................
4. Negli ultimi 12 mesi, ti sei preoccupato/a che a casa tua il cibo finisse prima di avere soldi per comprarne altro?<br>☐ Si, sempre ☐ No, mai<br>☐ Si, spesso<br>☐ Si, a volte	**0** 1 2 3 4 **5**	☐ **No**<br>☐ **Si, perché**<br>....................................
5. Pensa al posto in cui vivi: hai uno o più di questi problemi? (seleziona tutti quelli che hai)<br>☐ Infestazione da insetti<br>☐ Muffa<br>☐ Riscaldamento non sufficiente<br>☐ Forno o fornelli non funzionanti<br>☐ Perdite d'acqua<br>☐ Spazi insufficienti<br>☐ Ostacoli strutturali (gradini, mancanza di ascensore, bagno e doccia di difficile utilizzo, …)<br>☐ Altro<br>..	**0** 1 2 3 4 **5**	☐ **No**<br>☐ **Si, perché**<br>....................................
6. Sei preoccupata/o di non avere un alloggio stabile nei prossimi mesi?<br>☐ Si ☐ No	**0** 1 2 3 4 **5**	☐ **No**<br>☐ **Si, perché**<br>....................................

Responsabile *dott. Lorenzo Campedelli - cell. 324 152310 e-mail lorenzo.campedelli2@studio.unibo.it*

	↓ La domanda è chiara? ↓ 0 = Per niente chiara ↓ 5 = Chiarissima	↓ La domanda ti crea disagio?
7. Negli ultimi 12 mesi, l'azienda che ti fornisce elettricità, acqua o gas ti ha avvisato di voler interrompere la fornitura alla tua casa?<br>☐ Si ☐ No<br>☐ I servizi sono già stati interrotti	**0** 1 2 3 4 **5**	☐ **No**<br>☐ **Si, perché**<br>....................................
8. Ti è capitato di non avere abbastanza soldi per pagare le bollette?<br>☐ Si, sempre ☐ No, mai<br>☐ Si, spesso<br>☐ Si, a volte	**0** 1 2 3 4 **5**	☐ **No**<br>☐ **Si, perché**<br>....................................
9. Ti capita di rimandare o trascurare visite mediche o esami perché troppo lontane/i o perché hai difficoltà nei trasporti?<br>☐ Si ☐ No	**0** 1 2 3 4 **5**	☐ **No**<br>☐ **Si, perché**<br>....................................
10. Ti è successo di non poterti permettere spese mediche (visite mediche, dentista, medicine o trattamenti per la salute)?<br>☐ Si, sempre ☐ No, mai<br>☐ Si, spesso<br>☐ Si, a volte	**0** 1 2 3 4 **5**	☐ **No**<br>☐ **Si, perché**<br>....................................
11. Hai difficoltà ad avere aiuti nella cura dei bambini (asilo, scuola, baby-sitter)?<br>☐ Si ☐ No<br>***Se si,*** ti sta creando problemi nello studio o nel lavoro?<br>☐ Si ☐ No	**0** 1 2 3 4 **5**	☐ **No**<br>☐ **Si, perché**<br>....................................
12. Hai difficoltà nell'assistenza (care giving) di un familiare?<br>☐ Si ☐ No	**0** 1 2 3 4 **5**	☐ **No**<br>☐ **Si, perché**<br>....................................

Questionario per lo Screening dei Determinanti Sociali di Salute (DSS) in Cure Primarie

↓ **La domanda è chiara?** ↓ **La domanda ti crea disagio?**
↓ **0 = Per niente chiara** ↓
↓ **5 = Chiarissima** ↓

13. Se hai bisogno con attività della vita quotidiana, come lavarti, preparare i pasti, fare acquisti, gestire il denaro, ecc., riesci ad avere aiuto?
☐ Non ricevo nessun aiuto
☐ Ricevo tutto l'aiuto necessario
☐ Avrei bisogno di più aiuto
☐ Ho bisogno di molto più aiuto

0 1 2 3 4 **5**

☐ **No**
☐ **Si, perché**
....

14. Ti senti solo o isolato da chi ti circonda?
☐ Si, sempre ☐ No, mai
☐ Si, spesso
☐ Si, a volte

0 1 2 3 4 **5**

☐ **No**
☐ **Si, perché**
....

15. Se sei straniero: trovi difficoltà nell'inserimento tuo e della tua famiglia? (Iscrizione figli a scuola, imparare la lingua, avere permesso di soggiorno e documenti, richieste di bonus/agevolazioni, …)
☐ Si ☐ No

0 1 2 3 4 **5**

☐ **No**
☐ **Si, perché**
....

16. Quante volte ti capita che qualcuno, famigliari inclusi:

- ti faccia del male fisicamente?

☐ Mai
☐ Raramente
☐ A volte
☐ Abbastanza spesso
☐ Frequentemente

0 1 2 3 4 **5**

☐ **No**
☐ **Si, perché**
....

- ti prenda in giro o ti umili?

☐ Mai
☐ Raramente
☐ A volte
☐ Abbastanza spesso
☐ Frequentemente

0 1 2 3 4 **5**

☐ **No**
☐ **Si, perché**
....

Responsabile *dott. Lorenzo Campedelli - cell. 324 152310 e-mail lorenzo.campedelli2@studio.unibo.it*

Questionario per lo Screening dei Determinanti Sociali di Salute (DSS) in Cure Primarie

↓ **La domanda è chiara?** ↓ **La domanda ti crea disagio?**
↓ **0 = Per niente chiara** ↓
↓ **5 = Chiarissima** ↓

- minacci di farti del male?

☐ Mai
☐ Raramente
☐ A volte
☐ Piuttosto spesso
☐ Frequentemente

0 1 2 3 4 **5**

☐ **No**
☐ **Si, perché** ....

- urli contro di te o ti offenda con parolacce?

☐ Mai
☐ Raramente
☐ A volte
☐ Piuttosto spesso
☐ Frequentemente

0 1 2 3 4 **5**

☐ **No**
☐ **Si, perché** ....

17. Vorresti ricevere aiuto per qualcuno dei problemi elencati?

☐ Si ☐ No

Se si, indica il problema per cui vorresti ricevere aiuto:

☐ Istruzione (*domanda n.1*)
☐ Lavoro (*n. 2*)
☐ Spesa alimentare (*n. 3, 4*)
☐ Abitazione (*n. 5, 6*)
☐ Bollette (*n. 7, 8*)
☐ Sanità (*n. 9, 10*)
☐ Caregiving (*n. 11, 12*)
☐ Isolamento (*n. 13, 14*)
☐ Immigrazione (*n. 15*)
☐ Violenza (*n. 16*)

0 1 2 3 4 **5**

☐ **No**
☐ **Si, perché** ....

Hai qualche commento o suggerimento da darci sul questionario? Scrivi in libertà.

....
....
....
....
....
....

Se vuoi, puoi dirci anche:

Sei:
☐Maschio ☐Femmina

L'italiano è la tua lingua madre?
☐ Si ☐ No

A che fascia di età appartieni?
☐ < 15
☐ 15 - 35
☐ 36 - 55
☐ 56 - 74
☐ > 75

Grazie della tua partecipazione!

Responsabile *dott. Lorenzo Campedelli - cell. 324 152310 e-mail lorenzo.campedelli2@studio.unibo.it*

The EveryONE Social Needs Screening Tool of the American Academy of Family Physicians - English translation of the version translated and adapted to the italian language

DOMAINS	ITEM
EDUCATION	Do you have a high school degree?
EMPLOYMENT	Do you have a job?
FOOD	In the last 12 months, did it happen that you ran out of food at home and had no money to buy more?In the last 12 months, did you run out of food at your home before you had money to buy more?
HOUSING	Think about where you live: do you have one or more of these problems? Bug infestation,mold...-Are you worried about not having stable housing in the coming months?
UTILITIES	In the last 12 months, has the company supplying you with electricity, water or gas notified you that it wants to cut off the supply to your home?
FINANCES	Did you happen to not have enough money to pay your bills?
TRANSPORTATION	Do you find yourself postponing or neglecting doctor's visits or examinations because they are too far away or because you have transport difficulties?
*ACCESSIBILITY OF HEALTH CARE	Have you ever found yourself unable to afford medical expenses (doctor's visits, dentist, medicine or health treatment)?
CHILDCARE	Do you find it difficult to get help with childcare (kindergarten, school, babysitting)? If so, is it causing you problems in your studies or work?
*CAREGIVING	Do you find it difficult to care for (caregiving) a family member?
*SOCIAL SUPPORT	If you need help with activities of daily living, such as washing, preparing meals, shopping, managing money, etc., can you get help? - Do you feel lonely or isolated from those around you?
*IMMIGRATION	If you are a foreigner: do you find it difficult to integrate yourself and your family? (Enrolling your children in school, learning the language, getting a residence permit and documents, applying for bonuses/bonuses, ...)
PERSONAL SAFETY	How often does it happen that someone, including family members, physically hurts you? teases or humiliates you? threatens to hurt you ?shouts at you or swears at you?
ASSISTANCE	Would you like help with any of the problems listed?

Marked with * the items added from the research team to the original version of The EveryONE Social Needs Screening Tool of the American Academy of Family Physicians
